# Propolis Extract with Activity Against *Cutibacterium acnes* Biofilm Targeting the Expression of Virulence Genes

**DOI:** 10.3390/antiox14070849

**Published:** 2025-07-10

**Authors:** Sophia Athanasopoulou, Eleni Panagiotidou, Eleni Spanidi, Maria Gkika, Danai Georgiou, Athanasios K. Anagnostopoulos, Christos Ganos, Ioanna Chinou, Evangelos Beletsiotis, Konstantinos Gardikis

**Affiliations:** 1 Research and Development Department, APIVITA SA, Industrial Park Markopoulo Mesogaias, 19003 Athens, Greece; sofia.athanasopoulou@puig.com (S.A.); eleni.panagiotidou@puig.com (E.P.); eleni.spanidi@puig.com (E.S.);; 2 Biomedical Research Foundation, Academy of Athens, 11527 Athens, Greece; 3 Laboratory of Pharmacognosy and Chemistry of Natural Products, Department of Pharmacy, School of Health Sciences, National and Kapodistrian University of Athens, Zografou, 15771 Athens, Greece; 4 Molecular Microbiology Department, QACS The Challenge Test Laboratory, Antigonis 1, 14451 Metamorfosi, Greece; 5 Department of Pharmacy, Frederick University, Nicosia 1036, Cyprus

**Keywords:** propolis extract, *Cutibacterium acnes*, biofilm

## Abstract

Acne is a highly prevalent skin condition with multifactorial pathophysiology, where *Cutibacterium acnes* (*C. acnes*) overgrowths generate inflammation. *C. acnes* can grow and adhere, through the formation of biofilms, to almost any surface, which enables chronic infections. Acne treatment with antibiotics can induce topical antimicrobial resistance, impair microbiome biodiversity and cause cutaneous dysbiosis. In this study, we assess the effect of a standardized propolis extract (PE) from Greece against *C. acnes*, whilst maintaining skin’s microbiome biodiversity, and we investigate its effect against genes related to the attachment and colonization of *C. acnes*, as well as against biofilm formation. The extract has been chemically characterized by GC-MS and was additionally tested for its antioxidant properties by the Folin–Ciocalteu method and the 2,2-Diphenyl-1-Picrylhydrazyl (DPPH) assay and its regulatory activity on the expression of antimicrobial and anti-inflammatory genes in normal human epidermal keratinocytes (NHEKs). The suggested efficacy of PE in targeting pathogenic *C. acnes* biofilm, via downregulation of virulence genes, represents an alternative strategy to modulate the behavior of skin microbiota in acne, paving the way for next-generation acne-targeting products.

## 1. Introduction

Acne vulgaris is a highly prevalent skin condition with a multifactorial pathophysiology. The standard model suggests that follicular plugging occurs due to a sticky epithelium associated with increased sebum, resulting in anaerobic follicular conditions that favor *C. acnes* (formerly known as *Propionibacterium acnes*) bacterial overgrowth, generating inflammation [1]. Moreover, in the last decade many studies have consistently demonstrated that patients with acne experience increased oxidative stress [2].

The acne-causing strains of *C. acnes* inhabit skin-gland cavities and form sessile aggregates known as biofilms, causing inflammation and skin disorders [3]. Biofilms are structured consortia of microorganisms that attach to biotic or abiotic surfaces and are embedded within a self-produced extracellular matrix [4]. Biofilm formation is a multistep process in which microbial cells adhere to the surface (initial reversible adhesion), while the subsequent production of an extracellular matrix results in a more stable attachment. The biofilm of acne-associated *C. acnes* strains consists mainly of 62.6% polysaccharides, 9.6% proteins, 4.0% DNA and 23.8% other compounds (porphyrin precursors and others) [5]. The essential exogenous products secreted by *C. acnes* accumulate within the biofilm’s extracellular matrix, where they play a pivotal role in maintaining the structural and functional integrity of the biofilm. Immobilized (biofilm-associated) cells are phenotypically and physiologically different from non-adherent (planktonic) cells, and one of the typical properties of immobilized cells is their increased resistance to antimicrobial agents. It has been verified that *C. acnes* in biofilm is more resistant to antibiotics than bacteria in the planktonic growth phase [4]. *C. acnes* can form a biofilm both in vitro and in vivo. Biofilm formation is considered a primary factor contributing to the failure of antimicrobial therapies, with an estimated 65–80% of all infections involving biofilm-associated microbial communities [6,7].

The prevention of the formation of biofilms and action against the established biofilm is a strategy for the development of new anti-pathogenic treatments. An important role in the above actions is the obstruction of the ability of bacteria to adhere to the host [8].

The initial step in bacterial pathogenesis and biofilm formation involves the colonization of and attachment to host surfaces. Important factors in the fundamental stage of adhesion are bacterial virulence factors (secreted, cytosolic, or membrane-associated). These are bacterial molecules used by pathogens to colonize the host at the cellular level. Secreted bacterial factors play a critical role in evading both the innate and adaptive immune responses of the host. Cytosolic components contribute to metabolic, physiological, and morphological adaptations, whereas membrane-associated virulence factors facilitate bacterial adhesion to host tissues and biomaterial surfaces [9,10].

A number of natural compounds target bacterial virulence factors to disrupt pathogenic potential without affecting bacterial viability. An increasing line of evidence reveals propolis’ ability to interfere with virulence factor production and biofilm formation in multiple pathogenic microorganisms [11].

Propolis is a resinous substance produced by honeybees (*Apis melifera*). It exhibits multiple bioactive properties, including antimicrobial, immunostimulating, and antioxidant activities, and is utilized in the formulation of functional foods, cosmetics, and traditional medicinal applications [12,13,14,15,16]. Its composition varies depending on the geographic origin and flora thereof, and on the foraging area of the honeybees and the collection season. Propolis consists of about 50% resins, 30% waxes, 10% aromatic compounds, 5% pollen and 5% other ingredients [14,17,18]. The bioactive components of propolis are mostly phenolic compounds, terpenes, as well as sugars and amino acids [13,19,20,21,22]. Among the most abundant polyphenols are flavonoids, phenolic acids, as well as their esters. The structural complexity of propolis, coupled with its compositional variability, influenced by geographic origin and seasonal factors, results in different types of propolis, making it difficult or even impossible to determine the exact quality and formulate it into products for per os or cutaneous use intake (through the mouth) or products applied to the skin [23,24].

The antibacterial properties of propolis have been examined in a great number of Gram-positive and Gram-negative bacteria (>600 bacterial strains—reviewed in [15]) with variations in efficacy, depending on its composition and geographical origin. Propolis extracts either directly affect bacteria or stimulate the immune system of the host [25]. Gram-positive bacteria are more susceptible to propolis antibacterial activity, which affects bacterial mobility, disrupts their membrane potential, increases membrane permeability and decreases adenosine triphosphate (ATP) production [15].

Until today, very few studies have described the activity of propolis against *C. acnes*, while there are no reports of its activity against the biofilm of this bacterium. Recently the antioxidant, anti-inflammatory and anti-acne activities of stingless bee (*Tetragonula biroi*) propolis have been studied thoroughly, and have been reported to stimulate the growth of skin tissue, protect skin from photodamage and free radicals, prevent premature skin ageing, and help extinguish acne, especially for acne-prone skin [26].

The present study assesses the in vitro and ex vivo action of a specific Greek propolis extract on the microflora commonly found on human forehead skin, displays its effect on the formation and growth of biofilms and on genes related to the colonization ability of *C. acnes*, and demonstrates, at the cellular level, its ability to regulate the expression of antimicrobe- and anti-inflammation-associated genes in human keratinocytes.

## 2. Materials and Methods

### 2.1. Propolis Extraction

The 1.3 propanediol was supplied by Connect Chemicals (Vimercate (MB), Italy) while 3-[(2-ethylhexyl)oxy]propan-1,2-diol was provided by Ashland (Rotterdam, The Netherlands). Methanol was acquired by PanReac AppliChem (Darmstadt, Germany).

Raw propolis from Greek beekeeping activity from the wider area of the Olympus Mountain (Central Greece) was used. Typical plant families that bees use for propolis collection are *Asteraceae*, *Rosaceae*, *Liliaceae* and *Boraginaceae*, while they collect propolis from nectarless plants as well, such as *Quercus* sp., *Chenopodium* sp., *Scabiosa* sp., etc. [16]. The criterion was a concentration of total polyphenols >900 mg/L gallic acid equivalents (GAE) after dissolving 10% by weight of propolis in ethanol and subsequent measurement in a spectrophotometer using the Folin–Ciocalteu method.

The propolis was shredded into small particles (<1 mm) after cooling it for 24 h at −20 °C. It was then dispersed in deionized water at a temperature of 5–7 °C at a ratio of 1:1. The system was stirred under cooling for 15 min and then the supernatant floating impurities (wax, wood, bee parts) were discarded followed by filtration with filter paper under vacuum. The solid propolis as well as the water-dissolved propolis were then cooled for 24 h at −80 °C and then lyophilized under the following conditions: condenser temperature −50 °C, vacuum 8.2 × 10^−2^ mbar.

The wax-free propolis was again shredded into small pieces (<1 mm) after cooling for 24 h at −20 °C. It was then dispersed at a rate of 1 kg/min in the solvent system, which was under agitation at 1500 rpm. The concentration of propolis was 20% by weight. The extraction solvent system consisted of 1.3-Propanediol and the glycerylether ethylhexylglycerin (Ethylhexylglycerin) at a ratio of Ethylhexylglycerin:1.3-Propanediol: 3/97.

The resulting suspension was then placed in a pressure-assisted extractor (Timatic Micro, solid–liquid extractor, Tecnolab, Spello, Italy).

The additional extraction took place under a pressure of 6 bar and at a temperature of 25 °C. The process included 16 compression cycles, lasting 6 min and corresponding decompression cycles lasting 6 min. Then the mixture was left in a hermetically closed container at rest at 5–7 °C for 24 h. It was then filtered by an array of cartridge-type filters (Donaldson Filtration Deutschland GmbH, Haan, Germany) with a pore size of 10 μm–5 μm–1 μm–0.45 μm.

### 2.2. Quantification of Extract Components

Propolis crude material was extracted three times with 70% ethanol (1:10, *w*:*v*) by maceration at room temperature for 24 h, followed by filtration of the resulting suspension at room temperature using a paper filter and in-vacuum evaporation of the solvent to dryness on a rotary evaporator. For GC/MS analysis approximately 5 mg of each residue were silylated mixed with 40 μL of dry pyridine and 50 μL of BSTFA (bis(trimethylsilyl) trifluoracetamide) 25 mL for gas chromatography (CASno 25561-30-2) 2 CASno 25561-30-2 (Merck) and heated at 80 °C for 20 min [16].

### 2.3. GC-MS Analysis

The chemical analysis was performed by the technique of gas chromatography coupled with mass spectrometry (gas chromatography–mass spectrometry, GC-MS).

The analysis was performed on an Agilent 7820A gas chromatograph, connected to an Agilent 5977B mass spectrometer system (Agilent Technologies, Santa Clara, CA, USA) based on electron impact (EI) and 70 eV ionization energy. The gas chromatograph was also equipped with a split/splitless injector and a capillary column HP5MS 30 m, internal diameter 0.25 mm and membrane thickness 0.25 μm. The temperature was programmed from 100 to 300 °C at a rate of 5 °C/min. The carrier gas was He at a flow rate of 0.7 mL/min, injection volume of 2 μL, split ratio of 1:10 and injector temperature of 280 °C. The identification was accomplished using Wiley mass spectral databases and a database created by our research team [16].

### 2.4. Total Phenolic Content (TPC)

The quantification of total phenolic content was performed using the Folin–Ciocalteu reagent, adhering to the methodology outlined by Arnous et al. (2002) [27]. The phenols were determined using a calibration curve for 100 mg/L to 1200 mg/L of gallic acid (GA) and absorbance was 750 nm. The samples (0.02 mL) were diluted in ultra-pure water (1.58 mL) and the followed addition of the Folin-Ciocalteu reagent (0.1 mL) (Merck KGaA, Darmstadt, Germany) and mixed. After 1 min, aqueous Na_2_CO_3_ solution (20% *w*/*v*) (0.3 mL) was added and mixed by vortex. The mixture was kept for 2 h in darkness and at room temperature for 2 h, and absorbance measurement followed.

### 2.5. Antioxidant Activity via 2,2-Diphenyl-1-picrylhydrazyl (DPPH)

The antioxidant capacity of the colloidal systems was evaluated using the DPPH free radical assay, as detailed by Brand-Williams et al. (1995) [28]. PE (0.1 mL) was added to 3.9 mL of a 6 × 10^−5^ mol/L DPPH methanol solution. The decrease in absorbance was determined at 515 nm. The antioxidant activity was estimated from a standard curve of absorbance values derived from standard concentration solutions of Trolox.

Trolox Equivalents (mM) = 0.0177% DA_515nm_ + 0.08 R^2^ = 0.9998 (1)

where

% DA_515nm_ = Abs_515nm(t=0)_ − Abs_515nm(t=30)_/Abs_515nm(t=0)_ × 100 (2)

### 2.6. Stability of PE

PE was subjected to storage for two months under various conditions, including temperatures of 6 °C, 25 °C, 38 °C, and exposure to UV light, to assess its physical and chemical stability. The chemical stability was evaluated by measuring antioxidant activity using the DPPH free radical scavenging method and determining the total phenolic content with the Folin–Ciocalteu assay.

### 2.7. In Vitro Microbiome Preservation

The PE’s effect on the forehead microbiome was assessed with a three-phase in vitro/ex vivo test.

In phase 1, the influence on the growth behavior of microbes that comprise the majority of skin microbiome for this given area was examined on eight ATCC species, namely, *Staphylococcus epidermidis*, *Staphylococcus capitis*, *Cutibacterium acnes*, *Corynebacterium tuberculostearicum*, *Micrococcus luteus*, *Malassezia globosa*, *Staphylococcus hominis* and *Streptococcus mitis*. In phase 2, the effect on the growth behavior of specific pathogens was assessed on 12 ATCC species, namely, *Staphylococcus aureus*, *Pseudomonas aeruginosa*, *Klebsiella pneumoniae*, *Escherichia coli*, *Streptococcus mutans*, *Candida albicans*, *Candida glabrata*, *Malassezia furfur*, *Enterobacter cloacae*, *Streptococcus viridans*, *Candida tropicalis* and *Corynebacterium kroppenstedti*. For all microorganisms, an inoculum of 1 × 10^6^ CFU/mL was prepared and exposed for 2 h against the product (10% dilution). Their survival was then determined by serial dilutions in appropriate culture media. Finally, in phase 3, the effect of PE on the whole microbial biodiversity was examined by inoculating swabs taken from the forehead of two healthy volunteers (ex vivo). After a 2 h-exposure against the substance, the survival of the major groups [Total Aerobic Microbial count (Tryptic Soy Agar-TSA, Plate Count Agar-PCA, Brain Heart Infusion-BHI), Total Anaerobic Microbial count and *Staphylococcus* spp.] was determined by appropriate media and conditions.

### 2.8. Crystal Violet Staining for Quantitative Analysis of Biofilm

Quantification of biofilm was performed using a modified and optimized crystal violet assay method. Both the inhibitory effect on biofilm formation and the PE ability to eliminate established biofilms were assessed.

For the evaluation of the inhibition of biofilm formation, *C. acnes* strain ATCC 6919 was resuscitated and cultured for 72 h in tryptic soy broth (TSB) under anaerobic conditions. The culture was further diluted with TSB to an appropriate concentration (1 × 10^8^ CFU/mL) and 100 µL of this culture was inoculated into the wells of a sterile 96-well flat bottom plastic plate (Nunclon Surface F; Nunc A/S). The following conditions were examined: (i) biofilm positive control where only *C. acnes* was present, (ii) three different concentrations of the propolis substrate −0.1% *v*/*v*, 0.3% *v*/*v* and 0.5% *v*/*v*, (iii) biofilm negative control containing *C. acnes* along with 6 µg/mL of oxytetracycline and (iv) a sterility and background control containing only TSB. All wells had a final volume of 200 μL. Each concentration was tested in six technical replicates and the procedure was repeated twice. The microplate was incubated for 72 h under anaerobic conditions before biofilm staining. After incubation, the culture medium was removed, followed by gentle washing (three times) with 200 µL of phosphate-buffered saline (PBS). The plate was dried for 30 min at 45 °C and then stained with 200 μL of crystal violet (0.1% (*w*/*v*) in water) for 15 min. The stain was removed, and the wells were then washed three times with 200 µL of PBS. The dye was then extracted with 200 µL of acetone–ethanol (20:80 (*v*/*v*)) for 15 min. After the extraction, 100 µL were transferred to new wells and the absorbance was measured at 595 nm (OD_595_) in an ELISA reader (Biochrom EZ Read 400, Biochrom Ltd., Cambridge, UK). The absorbance of the negative control was subtracted from the absorbance of each well before statistical evaluation.

For the evaluation of the disruption of *C. acnes*-established biofilm, a biofilm of *C. acnes* was allowed to pre-form. Biofilm formation was achieved by spreading 100 μL of a standardized *C. acnes* culture in a 96-well microtiter plate. The microplate was incubated anaerobically at 37 °C for 3 days to allow cell attachment. The following conditions were examined: (i) biofilm positive control where only *C. acnes* was present, (ii) three different concentrations of the propolis substrate −0.1% *v*/*v*, 0.3% *v*/*v* and 0.5% *v*/*v*, (iii) biofilm negative control containing *C. acnes* along with 6 µg/mL of oxytetracycline and (iv) a sterility and background control containing only TSB. All wells had a final volume of 200 μL. The plate was further incubated for 48 h before the crystal violet staining was performed. The staining and cleaning were performed as outlined above.

The percentage of inhibition was determined through the Equation (3).

% Inhibition = [(OD negative control − OD sample)/OD negative control](3)

### 2.9. Gene Expression Analysis in C. acnes

The strain *C. acnes* ATCC6919 was resuscitated and cultured for 72 h in TSB under anaerobic conditions. The culture was then diluted with TSB under anaerobic conditions to an appropriate concentration (1 × 10^7^ CFU/mL) and incubated for 72 h under anaerobic conditions. Consequently, *C. acnes* cultures were exposed to PE at concentrations of 0.1% *v*/*v* for 24 h. For total RNA isolation as well as cDNA synthesis, Monarch^®^ Total RNA Miniprep Kit (Hitchin, UK) and PrimeScript-RT reagent Kit (Takara Bio, Otsu, Japan) were used, respectively. Equal amounts of cDNA were used for real-time polymerase chain reaction (qRT-PCR) and the expression of biofilm- and virulence-related genes *CAMP1*, *CAMP4*, *hyl*, *roxP* and *luxS* was analyzed. A 16 s rRNA gene was used as a housekeeping gene. The real-time polymerase chain reaction (qRT-PCR) method as well as the gene analysis procedure were performed according to the standard protocols of the Laboratory [29].

### 2.10. Gene Expression Analysis in Keratinocytes

NHEKs from an adult donor were purchased from Lonza (Basel, Switzerland) and cultured in KGM™ Gold medium, human epidermal growth factor, bovine pituitary extract, hydrocortisone, epinephrine, transferrin, and gentamicin/amphotericin Lonza Bioscience. The cells were left in culture until 70–80% fullness of the flask and then experiments were carried out with their 24-h incubations with the formulation (PE) at concentrations of 0.01% *v*/*v* and 0.03% *v*/*v*.

For total RNA isolation (500 ng) as well as cDNA synthesis, the Nucleospin RNA kit (Macherey-Nagel, Düren, Germany) and the PrimeScript-RT reagent kit (Takara Bio, Otsu, Japan) were used, respectively. Equal amounts of cDNA were used for real-time polymerase chain reaction (qRT-PCR) and the expression of *IL-4*, *DEF1B*, *ITGB2* and *CXCL12* genes was analyzed. The qRT-PCR method as well as the gene analysis procedure were performed according to the standard protocols of the Laboratory [29]. The glyceraldehyde 3-phosphate dehydrogenase gene (GAPDH) was chosen as a reference gene to normalize values. Two conditions are shown: NHEK cells not incubated with the active (N/T), and NHEK cells incubated with the compound (PE) at concentrations of 0.01% *v*/*v* and 0.03% *v*/*v*.

### 2.11. Statistical Analysis

Results are expressed as the mean ± SEM of two different experiments. For the in vitro microbiome preservation assay, all microbiological data were log-transformed (log10) before further processing. These were then tested for normality using the Shapiro–Wilk test. Variance homogeneity within groups was assessed using Levene’s test. When the assumption of homogeneity of variances was violated, Welch’s ANOVA was performed, followed by post hoc pairwise comparisons using the Games–Howell test (phase 1 and phase 2). In cases where the assumption of normality was not met, the Kruskal–Wallis test was applied, with Dunn’s test used for post hoc pairwise comparisons (phase 3). All data were processed on python v3.8.0 utilizing the NumPy, Pandas, SciPy, Matplotlib, Seaborn, Scikit-learn, Pingouin and Scikit-posthocs libraries. For all the other results, statistical analysis between two individual groups was performed by Student’s *t*-test and one-way ANOVA (Dunnett’s multiple comparisons test). A *p* ≤ 0.05 was considered statistically significant. Statistical analysis and graphs were performed with Sigma Plot Software v.10 and GraphPad Prism 9 (GraphPad software Inc., San Diego, CA, USA).

## 3. Results

### 3.1. Propolis Composition

The chemical composition and biological properties of propolis from different geographic regions are extremely valuable to solving the problem of different propolis types, as well as with their standardization (Table 1).

The different most common types of propolis as they are referred to in the literature [12] are (i) Poplar type (*Populus* spp. originated mainly from Europe, non-tropic regions of Asia, North America and New Zealand), (ii) Birch type (*Betula verrucosa* derived from Russia), (iii) Green type (*Baccharis* spp. mostly characteristic of Brazil), (iv) Red type (*Dalbergia* spp. which is located in Brazil, Mexico and Cuba), (v) Clusia type (from *Clusia* spp. from Venezuela and Cuba), (vi) Pacific type (*Macaranga tanarius* originated from Indonesia, Taiwan and Okinawa), and (vii) Mediterranean type (plants mainly from Cupressaceae family which is located in Greece, Sicily, Malta and other Mediterranean countries).

Of the main chemical classes of the identified compounds (Table 1) in all samples, the most abundant type of metabolites were the flavonoids and chalcones (27.80%), followed by aromatic acid/hydrocarbon (22.30%), aliphatic acid/ether (15.30%) and esters of aromatic acids (11.40%), respectively.

In the current study, the chemical profile of the propolis sample, as captured by the GC/MS method, identified the existence of high content in phenolic compounds (flavonoids and chalcones together with aromatic acids and their esters). Moreover, among the identified flavonoids/chalcones, methoxyflavones, chalcones, galangin, dihydroxy-methoxyflavone, pinocembrin, pinobanksin and its 3-O-acetate, as well as caffeic and ferulic acids and their derivatives are characterized in higher percentages, which are typical constituents representing the European type of propolis [13,16].

The antioxidant activity measured 31.55 ± 1.55 mM Trolox equivalents, and the initial total phenol concentration was 7046 ± 394 μg GA/mL. Throughout a 6-week period, both the antioxidant capacity and total polyphenol levels remained stable, showing no significant variations (Table 2).

### 3.2. Sensitivity of Skin Microbiome to PE In Vitro and Ex Vivo

To test, in vitro, whether the PE preserves the skin microbiome, we selected a panel of bacteria species that are commonly found in the forehead (normal flora). The panel consisted of *Staphylococcus epidermidis*, *Staphylococcus capitis*, *Cutibacterium acnes*, *Corynebacterium tuberculostearicum*, *Micrococcus luteus*, *Malassezia globosa*, *Staphylococcus hominis* and *Streptococcus mitis* (Table 3). The bacteria were exposed to three different concentrations of PE for 2 h and enumerated on appropriate culture media after the exposure. The results are shown in Table 3. Furthermore, a second panel of 12 pathogenic bacteria was also exposed to PE (Table 4). At a concentration of 0.1% *v*/*v*, PE did not affect the viability of the tested bacteria. At a concentration of 0.3% *v*/*v*, the population of *C. acnes* and *S. mitis*, part of the normal flora, as well as the population of *Candida glabrata* and *Streptococcus viridans*, part of the pathogenic flora, were significantly reduced. Finally, at the concentration of 0.5% *v*/*v*, PE negatively affected the survival of four microorganisms of the normal flora panel and the survival of five of the pathogenic panel. However, all microbiological differences in abundance were considered ‘statistically non-significant’ in the multiple pairwise comparisons, regardless of the panel or PE concentration (Tables S1 and S2 in Supplementary Materials), indicating that there are no harmful effects in the panel of the tested microorganisms.

For the ex vivo testing, swabs from the forehead of two healthy volunteers were inoculated and exposed to 0.1% *v*/*v*, 0.3% *v*/*v* and 0.5% *v*/*v* PE for 2 h and the survival of total anaerobic flora, total aerobic flora and *Staphylococcus* spp. was evaluated. According to the results presented in Table 5, the concentration 0.1% *v*/*v* PE had no impact on the microbial biodiversity, at concentration 0.3% *v*/*v* PE slightly affected the total aerobic flora, while at 0.5% *v*/*v*, PE reduced the levels of total aerobic flora. Nonetheless, these effects were found to be ‘statistically non-significant’ (Table S3 in Supplementary Materials).

### 3.3. PE Inhibits Biofilm Growth and Development and Alters the Gene Expression Levels of C. acnes

**Figure 1:**
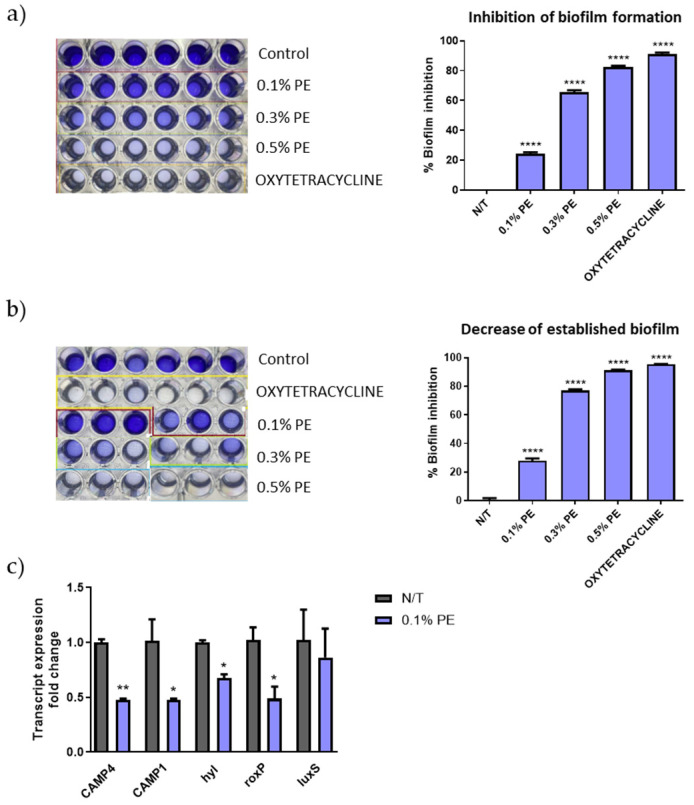
Biofilm disruption and altered gene expression of *C. acnes* by PE. Dose-dependent (**a**) inhibition of biofilm formation and (**b**) decrease of established biofilm as seen by crystal violet staining. The experiment was assessed in two independent replicates. Purple color intensity reflects biofilm biomass. (**c**) Relative gene expression levels for *CAMP4*, *CAMP1*, *hyl*, *roxP* and *luxS* of *C. acnes* cells exposed to 0.1% *v*/*v* PE expressed as a fold change ± SEM compared to untreated N/T. The experiment was assessed in two independent replicates. Differences were analyzed using one-way ANOVA with Dunnett’s test (**a**,**b**) or the unpaired Student’s *t* test (**c**). **** *p* < 0.001, ** *p* < 0.01, * *p* < 0.05.

*C. acnes* strains are classified into phylotypes IA1, IA2, IB1, IB2, IB3, IC, II and type III, with types IA1 and IA2 being predominantly found in acne lesions [30]. The *C. acnes* strain ATCC 6919, a strain isolated from facial acne and corresponding to phylotype IA1, was used for the estimation of the antibiofilm activity of PE extract. PE significantly inhibited the biofilm formation (Figure 1a) and decreased the established biofilm (Figure 1b) in a concentration-analogous manner. Interestingly, PE at the concentration of 0.5 *v*/*v*% inhibited biofilm formation at a similar percentage with that of tetracycline that was used as positive control (89.07% and 92.12% respectively). Furthermore, qPCR results revealed a decrease in gene expression of the putative virulence factors *CAMP1*, *CAMP4*, *roxP* and *hyl* (Figure 1c).

### 3.4. PE Alters the Gene Expression in Keratinocytes

Treatment of NHEKs with PE for 24 h resulted in significant alterations in the expression of genes responsible for a host’s antimicrobial response and anti-inflammatory action. Specifically, qPCR analysis demonstrated that *IL-4* gene expression was upregulated up to 55-fold, indicating a robust response to the compound. *DEF1B* expression increased by up to 6-fold.

**Figure 2:**
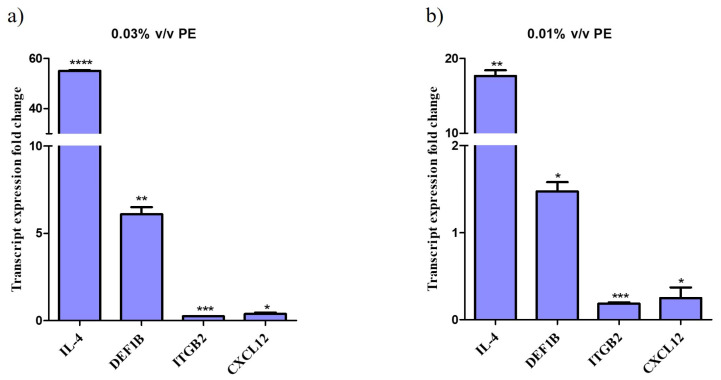
Altered gene expression profile of keratinocytes treated with PE. Relative gene expression levels for *IL-4*, *DEF1B*, *ITGB* and *CXCL12* of keratinocytes exposed to (**a**) 0.01% *v*/*v* and (**b**) 0.03% *v*/*v* PE expressed as a fold change ± SEM compared to untreated N/T. The experiment was assessed in two independent replicates. Differences were analyzed using one-way ANOVA with Dunnett’s test. **** *p* < 0.0001, *** *p* < 0.001, ** *p* < 0.01, * *p* < 0.05.

Conversely, the expression of *ITGB2* was downregulated by up to 5-fold and *CLCX12* by up to 4-fold. The data indicate a dose-response relationship, with significant upregulation observed at higher PE concentrations compared to the control (not-treated cells). Graphs depicting the fold changes in gene expression are shown in Figure 2.

## 4. Discussion

This study provides an analysis of the bioactivity of a Greek propolis extract. Specifically, we investigated the dose-dependent bioactivity of the extract against pathogenic constituents of the human forehead skin microbiota, while ensuring the preservation of the area’s microbial biodiversity. Furthermore, in the case of *C. acnes*, the PE downregulated the expression of key genes involved in attachment, colonization, and biofilm formation. Subsequent analyses confirmed that PE significantly inhibited both biofilm development and the growth of *C. acnes*. Finally, PE was found to modulate the expression of genes associated with antimicrobial defense and anti-inflammatory responses in NHEKs.

In this study we reveal for the first time the inhibitory properties of propolis on *C. acnes* biofilm. Biofilm formation is a well-documented strategy used by many bacterial species to enhance resistance to antibiotics [31]. The formation of biofilm is a multi-stage process where microbial cells first attach to a surface (initial reversible adhesion), followed by the production of an extracellular matrix that strengthens the attachment. The biofilm can negatively affect the entry of antibiotics and is a potent contributing factor to *C. acnes* resistance and failure of treatments, thus biofilm is a promising target for therapeutic strategies that target *C. acnes* [32]. Biofilm inhibition can occur through multiple mechanisms targeting different stages of biofilm development. These include preventing the initial bacterial adhesion to surfaces, interfering with quorum sensing communication pathways essential for biofilm formation, and disrupting the extracellular matrix that stabilizes the biofilm structure. Additionally, biofilm inhibition may involve the direct killing or growth inhibition of sessile cells within the biofilm, as well as impeding biofilm maturation and maintenance processes. Various natural and synthetic compounds have demonstrated efficacy in these roles, often acting through a combination of antimicrobial activity, anti-adhesive effects, and signaling interference, making them promising candidates for controlling biofilm-associated infections [33].

Various propolis extracts have shown antimicrobial activity against pathogenic microorganisms, such as bacteria of the genus *Salmonella* spp., but also against microorganisms found in the microflora of the skin (microbiome), such as bacteria *Staphylococcus epidermidis* [15]. Considering the contribution of the microbiome to the integrity of the skin, products intended for direct application to the skin that aim to inhibit pathogenic or potentially (opportunistically) pathogenic microorganisms, should not affect the qualitative and quantitative composition of the commensal microbiome.

Antibiotics, which have long been a staple of acne therapy, can cause localized antimicrobial resistance, inadvertently affect beneficial microbial communities and induce cutaneous dysbiosis—a disruption in the natural balance of the skin microbiome [3]. Moreover, antibiotics’ impact on microbial diversity can weaken the skin’s barrier function, making it more susceptible to irritation and infection. Reduced microbial diversity has been linked to other dermatological conditions, such as dermatitis or secondary infections [3,34].

PE was characterized as microbiome-preserving regarding normal microflora. At the tested concentrations of 0.1% *v*/*v* and 0.3% *v*/*v* demonstrated in vitro and ex vivo, it leaves the natural biodiversity of the forehead area unaffected. Interestingly, when tested at the concentration of 0.5% *v*/*v*, PE showcased a “non-friendly” behavior towards commensal microflora, as it significantly altered the forehead’s microbiome diversity, and multiple species’ growth was affected.

RoxP, uniquely produced by *C. acnes*, plays a crucial role in shaping the lipidome and, consequently, constitutes a significant component of the interaction interfaces between the host and microbes [35,36]. Although RoxP has been suggested to possess antioxidant properties and maintain redox homeostasis in normal skin, there is no data concerning its actual role in inflammatory conditions such as acne. We observed a significant reduction in gene expression levels of *roxP* upon treatment of *C. acnes* with PE. Our data support the idea that *roxP* may be a target for potent therapeutic interventions regarding this specific skin condition, worthy of further investigation. Apart from *roxP*, PE treatment reduced the gene expression levels of *hyl*. Hyl has been reported to be involved in the spread of bacteria [37]. Furthermore, PE significantly reduced the mRNA levels of the virulence factors CAMP1 and CAMP4, that can trigger inflammation in host tissue [38].

The bioactivity of PE was further assessed on human keratinocytes, focusing on the transcriptomic regulation of genes related to antimicrobial response—as propolis is known also for stimulating the host’s immune system [15,39]. We observed a significant transcriptional upregulation of interleukin-4, an pleiotropic cytokine that has positive effects on the outcome of infectious diseases, contributing to tissue healing [40,41], and of B-defensin 1 (DEF1B), a factor that exhibits microbicidal activity mainly against Gram-negative bacteria by interfering with the synthesis of their cell wall and causing microbial death in a manner similar to that of antibiotics [42]. Moreover, qPCR results showed a downregulation of β2-integrins (ΙΤGB2), receptors that are exploited by common skin pathogens to establish contact with host cells [43,44], and of C-X-C ligand motif chemokine 12 (CXCL12 or SDF-1), a chemokine with an important role in skin inflammation and inflammatory angiogenesis [45,46].

Collectively, our data highlight that this dual mechanism of PE—targeting biofilm-forming pathogenic strains of *C. acnes*, via direct inhibition of *C. acnes* virulence factors and enhancement of the host’s antimicrobial defenses—provides a multifaceted approach to modulate the behavior of skin microbiota in acne, paving the way for next-generation acne-targeting products. Aiming to investigate further the efficacy of PE in vivo, we are in the process of the incorporation of PE into three types of formulas (a cream, a cleansing gel and a lotion), that will be tested on volunteers with acne-prone skin. With respect to skin microbiota, in vivo studies are designed to evaluate the sensitivity of skin microbiota biodiversity using advanced sequencing techniques. These results will provide additional insights into the use of the PE on acne-targeting products.

## 5. Conclusions

Our findings highlight that this dual approach—targeting biofilm-forming pathogenic strains of *C. acnes* through direct inhibition of its virulence factors and boosting the host’s antimicrobial defenses—offers a comprehensive strategy to influence the behavior of skin microbiota in acne.

## Figures and Tables

**Table 1:** Chemical composition and relative concentration (area%) of the studied propolis sample.

No	RT (min)	Compound	Area%	Chemical Type of Compounds
1	5.40	Ethyl phenyl ether	4.90	Ether
2	6.40	Benzoic acid	9.70	Aromatic acid
3	7.16	Butanedioic acid	10.40	Aliphatic acid
4	9.23	Dimethyl-phenanthrene	12.60	Aromatic hydrocarbon
5	9.81	unknown	2.50	-
6	10.28	γ-curcumene	0.64	Sesquiterpene
7	10.34	ar-curcumene	1.27	Sesquiterpene
8	12.79	calarene	1.30	Sesquiterpene
9	13.49	cadinene	0.56	Sesquiterpene
10	13.86	β-eudesmol	0.82	Sesquiterpene
11	14.24	longifolene	0.91	Sesquiterpene
12	17.89–20.51	sugars	6.70	Sugars
13	27.38	ferulic acid ester	0.65	Ester of Aromatic acid
14	28.58	unknown	1.24	-
15	29.18	unknown	1.66	-
16	29.3	pinostrobin chalcone	0.37	Flavonoids/Chalcones
17	29.62	coumarate/caffeate derivative	0.73	Ester of Aromatic acid
18	29.85	pinocembrin chalcone	0.96	Flavonoids/Chalcones
19	29.95	pinocembrin	0.80	Flavonoids/Chalcones
20	30.41	caffeate derivative	0.74	Ester of Aromatic acid
21	30.51	hydroxy methoxy chalcone	5.38	Flavonoids/Chalcones
22	30.9	pinobanksin	3.32	Flavonoids/Chalcones
23	31.6	methoxylflavanone	5.13	Flavonoids/Chalcones
24	32.09	pinobanksin-3-acetate	2.58	Flavonoids/Chalcones
25	32.52	chalcone	3.85	Flavonoids/Chalcones
26	33.18	galangin	2.71	Flavonoids/Chalcones
27	33.68	phenyl ethyl ester of caffeic acid	1.80	Ester of Aromatic acid
28	33.88	dihydroxymethoxyflavone	0.77	Flavonoids/Chalcones
29	34.08	caffeic acid derivative	1.70	Ester of Aromatic acid
30	35.96	cinnamyl ester of isoferulic acid	1.67	Ester of Aromatic acid
31	36.85	cinnamyl ester of caffeic acid	311	Ester of Aromatic acid
32	37.3	alpinon chalcone	1.03	Flavonoids/Chalcones
33	37.46	kaempferol derivative	0.90	Flavonoids/Chalcones
34	40.17	unknown	1.76	-
35	42.32	unknown	2.24	-
		**Total**	**98.40**	

**Table 2:** Evaluation of stability test of DPPH: Antioxidant activity (±SEM) and TPCs (±SEM) of the PE.

Time	Conditions	TPC (μg GA/mL)	DPPH (mM Trolox Equivalent)
T (0)		7046 ± 227	31.55 ± 0.89
2 months	RT	8309 ± 63.5 **	36.84 ± 0.61 *
	6 °C	5782 ± 271.6 **	26.59 ± 1.37 *
	38 °C	6666 ± 176.2	29.01 ± 1.45
	UV	6611 ± 145.5	28.70 ± 0.73

Differences compared to T0 were analyzed using one-way ANOVA ** *p* < 0.01, * *p* < 0.05.

**Table 3:** Microbial count of bacteria species commonly found in the forehead after exposure to various concentrations of PE. Results are shown as log (CFU/mL).

Microorganisms	Control (0 h) (Mean ± SEM)	0.1% *v*/*v* PE (2 h) (Mean ± SEM)	0.3% *v*/*v* PE (2 h) (Mean ± SEM)	0.5% *v*/*v* PE (2 h) (Mean ± SEM)
*Staphylococcus epidermidis*	7.24 ± 0.05	7.31 ± 0.02	6.97 ± 0.08	6.34 ± 0.09
*Staphylococcus capitis*	7.79 ± 0.18	7.31 ± 0.04	7.80 ± 0.09	7.77 ± 0.12
*Cutibacterium acnes*	6.70 ± 0.10	6.74 ± 0.06	5.24 ± 0.04 ^#^	1.70 ± 0.00 ^#^
*Corynebacterium tuberculostearicum*	5.91 ± 0.04	5.93 ± 0.11	5.32 ± 0.04	3.02 ± 0.22 ^#^
*Micrococcus luteus*	7.03 ± 0.05	6.92 ± 0.02	6.88 ± 0.07	6.92 ± 0.03
*Malassezia globosa*	6.08 ± 0.01	6.03 ± 0.02	5.66 ± 0.08	1.70 ± 0.00 ^#^
*Staphylococcus hominis*	6.56 ± 0.07	6.57 ± 0.09	6.44 ± 0.10	5.89 ± 0.08
*Streptococcus mitis*	5.65 ± 0.07	4.89 ± 0.01	4.02 ± 0.06 ^#^	1.70 ± 0.00 ^#^

^#^ Reduction > 1 log.

**Table 4:** Microbial count of pathogenic bacteria species after exposure to various concentrations of PE. Results are shown as log (CFU/mL).

Microorganisms	Control (0 h) (Mean ± SEM)	0.1% *v*/*v* PE (2 h) (Mean ± SEM)	0.3% *v*/*v* PE (2 h) (Mean ± SEM)	0.5% *v*/*v* PE (2 h) (Mean ± SEM)
*Staphylococcus aureus*	7.82 ± 0.12	7.61 ± 0.02	7.34 ± 0.04	6.94 ± 0.06
*Pseudomonas aeruginosa*	8.08 ± 0.12	8.02 ± 0.08	7.89 ± 0.09	7.74 ± 0.16
*Klebsiella pneumoniae*	7.95 ± 0.02	8.18 ± 0.01	8.11 ± 0.04	8.07 ± 0.05
*Escherichia coli*	7.95 ± 0.05	8.16 ± 0.04	8.05 ± 0.11	8.01 ± 0.03
*Streptococcus mutans*	5.86 ± 0.08	5.78 ± 0.03	5.59 ± 0.02	5.55 ± 0.05
*Candida albicans*	6.47 ± 0.08	6.51 ± 0.05	6.10 ± 0.00	5.72 ± 0.10
*Candida glabrata*	6.27 ± 0.13	6.09 ± 0.12	4.96 ± 0.20 ^#^	3.48 ± 0.12 ^#^
*Malassezia furfur*	7.32 ± 0.03	7.16 ± 0.04	7.01 ± 0.07	5.36 ± 0.07 ^#^
*Enterobacter cloacae*	8.35 ± 0.04	8.41 ± 0.01	8.33 ± 0.05	8.41 ± 0.06
*Streptococcus viridans*	6.26 ± 0.01	4.92 ± 0.15 ^#^	3.70 ± 0.18 ^#^	3.60 ± 0.13 ^#^
*Candida tropicalis*	5.92 ± 0.10	5.82 ± 0.06	5.50 ± 0.22	4.35 ± 0.05 ^#^
*Corynebacterium kroppenstedtii*	6.32 ± 0.01	6.30 ± 0.01	5.81 ± 0.12	1.70 ± 0.00 ^#^

^#^ Reduction > 1 log.

**Table 5:** Microbial count of bacteria isolated from the forehead of two healthy volunteers and exposed to various concentrations of PE. Results are shown as log (CFU/mL).

Microorganisms	Control (0 h) (Mean ± SEM)	0.1% *v*/*v* PE (2 h) (Mean ± SEM)	0.3% *v*/*v* PE (2 h) (Mean ± SEM)	0.5% *v*/*v* PE (2 h) (Mean ± SEM)
Total Aerobic Microbial count (TSA)	4.31 ± 0.74	3.90 ± 0.45	3.36 ± 0.28 ^#^	3.00 ± 0.04 ^#^
Total Aerobic Microbial count (PCA)	4.69 ± 0.44	4.42 ± 0.38	4.14 ± 0.31	3.74 ± 0.09 ^#^
Total Aerobic Microbial count (BHI)	4.58 ± 0.45	4.16 ± 0.40	3.89 ± 0.26	3.65 ± 0.13 ^#^
Total Anaerobic Microbial count (RCA)	6.41 ± 0.11	6.45 ± 0.30	6.34 ± 0.16	6.38 ± 0.26
Growth medium for *Staphylococcus* (MSA)	4.79 ± 0.44	4.43 ± 0.45	4.21 ± 0.24	3.93 ± 0.21

^#^ Reduction > 1 log.

## Data Availability

The data presented in this study are available upon request from the corresponding author.

## Supplementary Materials

The following supporting information can be downloaded at: https://www.mdpi.com/article/10.3390/antiox14070849/s1.
